# Pregnancy and COVID-19: prevention, vaccination, therapy, and beyond

**DOI:** 10.3906/sag-2106-134

**Published:** 2021-12-17

**Authors:** Dilek ŞAHİN, Atakan TANAÇAN, Sophia NE WEBSTER, Özlem MORALOĞLU TEKİN

**Affiliations:** 1 Department of Obstetrics and Gynecology, Ankara City Hospital, Ankara Turkey; 2 Department of Obstetrics and Gynecology, Newcastle-Upon-Tyne Hospital, Newcastle United Kingdom; 3 Member of COVID-19 Scientific Advisory Board of Ministry of Health

**Keywords:** COVID-19, obstetric complications, pregnancy, SARS-CoV-2, vaccines

## Abstract

Coronavirus disease 2019 (COVID-19) caused by severe acute respiratory syndrome coronavirus 2 (SARS-CoV-2) has alarmed the world since its first emergence. As pregnancy is characterized by significant changes in cardiovascular, respiratory, endocrine, and immunological systems, there are concerns on issues like the course of disease in pregnant women, safety of medications, route of delivery and risk of obstetric complications. The aim of this review is to summarize the current literature in the management of pregnant women during the COVID-19 pandemic. Although more than 90% of pregnant women with COVID-19 recover without serious morbidity, rapid deterioration of disease and higher rates of obstetric complications may be observed. The risk of vertical transmission has not been clearly revealed yet. Decreasing the number of prenatal visits, shortening the time allocated for the examinations, active use of telemedicine services, limiting the number of persons in healthcare settings, combining prenatal tests in the same visit, restricting visitors during the visits, providing a safe environment in healthcare facilities, strict hygiene control, and providing personal protective equipment during the visits are the main strategies to control the spread of disease according to current guidelines. Although new medication alternatives are being proposed every day for the treatment of COVID-19, our knowledge about the use of most of these drugs in pregnancy is limited. Preliminary results are promising for the administration of SARS-CoV-2 vaccines in the pregnant population. Timing of delivery should be decided based on maternal health condition, accompanying obstetric complications and gestational age. Cesarean delivery should be performed for obstetric indications. Breast feeding should be encouraged as long as necessary precautions for viral transmission are taken. In conclusion, an individualized approach should be provided by a multidisciplinary team for the management of pregnant women with COVID-19 to achieve favorable outcomes.

## 1. Introduction

Coronavirus disease 2019 (COVID-19) caused by severe acute respiratory syndrome coronavirus 2 (SARS-CoV-2) has alarmed the world since its first emergence [1]. It significantly changed people’s daily routines, forcing countries to make comprehensive regulations. Social isolation, strict hygiene control, remote working, lockdown, and comprehensive immunization programs are the main strategies to control the pandemic in this extraordinary period [2]. However, it has been more than a year since the beginning of the pandemic and the disease has not been fully controlled yet. During this period, healthcare professionals made a superhuman effort and unfortunately some of them lost their lives during this tough process [3].

Like other branches of medicine, obstetrics have been also affected by the pandemic. During this period, health authorities had to make some important regulations to protect mothers, babies, and healthcare professionals from COVID-19 [4]. Reducing the number of antenatal visits, encouraging telemedicine services, combining routine screening tests in the same session, and providing adequate personal protective equipment are some of the methods to control the spread of infection during the pandemic [4]. As pregnancy is characterized by significant changes in cardiovascular, respiratory, endocrine, and immunological systems, there are concerns on issues like the course of disease in pregnant women, safety of medications, route of delivery and risk of obstetric complications [5]. Our experience on these issues is increasing day by day and management guides are regularly updated in this context Coronavirus infection and pregnancy Version 7 2020: updated 09/04/2020. Website https://www.rcog.org.uk/coronavirus-pregnancy [accessed on 31 May 2021],^2 ^ACOG. Novel Coronavirus 2019: updated 1.5.2021. Website https://www.acog.org/clinical/clinical-guidance/practice-advisory/articles/2020/03/novel-coronavirus-2019 [ accessed on 31 May 2021],^3^ Turkish Ministry of Health, General Directorate of Public Health, COVİD-19 Guideline, Scientific Committee Report: updated 21.08.2020. https://covid19bilgi.saglik.gov.tr/depo/rehberler/COVID-19_Rehberi.pdf?type=file [accessed on 31 May 2021]. 

The aim of this review is to summarize the current literature in the management of pregnant women during the COVID-19 pandemic.

## 2. Epidemiology and virology 

At the end of 2019, a series of pneumonia cases were reported in Wuhan, a city in the Hubei Province of China. Investigations revealed a novel coronavirus responsible for the mentioned infection and it rapidly spread all over the globe resulting in a pandemic [6]. Since its emergence, SARS-CoV-2 has infected millions of people leading to significant morbidity and mortality [6]. 

Coronaviruses are enveloped positive-stranded RNA viruses. SARS-C0V-2 is a betacoronavirus sharing the same subgenus with the severe acute respiratory syndrome (SARS) and the Middle East respiratory syndrome (MERS) viruses [7]. It enters the host cell via angiotensin-converting enzyme 2 (ACE2) receptor. SARS-CoV-2 binds to ACE2 receptor with its spike protein and the cellular protease transmembrane protease, serine 2 (TMPRSS2) has a significant role in this process [8]. Several mutations in the genome of SARS-CoV-2 have been observed since the beginning of the pandemic. The mutations mainly resulted in a functional change in the spike protein. Although most of them have no clinical significance, some of them have a potential to rapidly infect a great number of cases [9]. The most popular defined variants are B.1.1.7 lineage (first identified in the United Kingdom), B.1.351 lineage (first identified in South Africa), P.1 lineage (first identified in Japan in four travelers from Brazil), B.1.427/ B.1.429 lineages (first identified in the Southern California) and B.1.617 lineage (first identified in India) [10].

## 3. Prevention

The main mode of SARS-CoV-2 transmission is close person-to-person contact. Respiratory secretions are considered the source of viral transmission, and inhalation of viral particles or contact with mucous membranes cause transmission of infection [11]. SARS-CoV-2 is also detected in stool, blood, ocular secretion, and semen samples. However, their roles in the transmission of disease are uncertain [12]. The risk of transmission starts even in the asymptomatic period and it is highest in the earlier days of the disease. The risk of transmission significantly decreases after 7 to 10 days of illness [13]. Viral load, duration of contact, and use of personal protective equipment are all important factors for the risk of transmission [14].

The main strategy for controlling the spread of COVID-19 is preventing close contact with an infected individual. For this reason, social distancing, remote working, establishment of telehealth services, application of distance learning programs, lockdown, strict hygiene control, screening high-risk populations, administration of filiation programs, self-quarantine and providing broad-based use of personal protective equipment have been widely used by governments during the pandemic [15].

Pregnant women should also pursue the mentioned life-style changes in order to reduce the risk of transmission. They should effectively use personal protective equipment and special care should be taken for pregnant women with children in the home. Working conditions should be arranged according to underlying comorbidities and workplace environment [4,16]. 

There are other issues in the prevention of disease like vaccination and pre-/postexposure prophylaxis [17,18]. However, the efficacy of pre-/postexposure prophylaxis has not been proven yet, and the safety of vaccines in pregnant women is still under investigation [18,19]. The role of vaccination in pregnant women will be discussed in detail later in the present review.

Another important problem is the reproductive decision-making during the pandemic period. Although the course of COVID-19 seems to be worse in pregnant women, the potential pregnancy-related risks associated with SARS-CoV-2 have not been fully clarified yet [20]. Thus, pregnancy can be planned by taking necessary precautions. 

## 4. Clinical findings

Clinical findings in pregnant women with COVID-19 are similar to those in nonpregnant population [21]. According to the results of a study including 461,825 pregnant women with laboratory-confirmed infection, the most common symptoms were: cough (50.3%), headache (42.7%), muscle aches (36.7%), fever (32.0%), sore throat (28.4%), shortness of breath (25.9%), and loss of taste or smell (21.5%) [21]. A preliminary study including 29 confirmed and 71 clinically suspected pregnant women with COVID-19 from Turkey reported cough (58.6%) and myalgia (51.7%) as the leading symptoms [22]. Updated version of the mentioned study including 533 confirmed cases reported cough (33.4%) and myalgia (31.5%) as the leading symptoms again [23]. Nausea, vomiting, fatigue, diarrhea, and rhinorrhea may also accompany COVID-19 to a lesser extend [21–23].

The rate of asymptomatic cases was investigated in various studies [24–26]. A prospective study from Turkey including 206 pregnant women (103 low-risk pregnant women without any defined risk factor and 103 high-risk pregnant women) reported positive real-time reverse transcriptase polymerase chain reaction (RT-PCR) results in 3 cases (1.4%). All of them were in the high-risk pregnancy group [24]. A systematic review including 77 studies performed on 11,432 pregnant women reported a positivity rate of 7% in the universal screening and 73% of them were asymptomatic. Additionally, the mentioned study claimed that pregnant women were more likely to be asymptomatic than nonpregnant people of reproductive age with COVID-19 [25]. Another systematic review revealed that 95% of pregnant women with COVID-19 were asymptomatic [26]. 

## 5. Radiologic imaging and laboratory findings

Like other viral pathogens SARS-CoV-2 may cause increased levels of acute phase reactants, leukocyte count, and liver enzymes together with decreased levels of lymphocyte and platelet counts. According to the results of a systematic review, increased C-reactive protein (CRP) levels (49%), lymphopenia (33%), leukocytosis (26%), elevated procalcitonin levels (23%), abnormal liver enzymes (15.4%), and thrombocytopenia (6.6%) were the most common laboratory findings [28]. Lymphopenia (30.4%), anemia (8.4%), elevated liver enzymes (4.5%), and hypokalemia (1.5%) were the most significant findings in a comprehensive study from Turkey [23]. 

According to the results of a systematic review including 427 pregnant women with COVID-19, ground-glass opacities (77%), posterior lung involvement (73%), multilobar involvement (72%), bilateral lung involvement (69%), peripheral distribution (68%), and consolidation (41%) were the most common pathologic findings in the computerized tomography (CT) [27]. Radiologic imaging was performed in 12.7% of the pregnant women, and 57.3% of them were suspicious for COVID-19 according to the results of a prospective study from Turkey [23].

Physicians should keep in mind that physiological changes during pregnancy and sometimes pregnancy complications like preeclampsia may also cause alterations in laboratory tests. Thus, individualized approach should be applied for all cases and every pregnant woman should be evaluated rigorously to provide optimal healthcare. Another important factor is the concern of pregnant women for possible adverse effects of radiation on their babies. However, it has been long known that a radiation dose less than 0.05 gray has no known adverse effect on the growing fetus and chest imaging may be performed safely providing abdominopelvic protection [28]. Furthermore, lung ultrasonography is a safer alternative in selected cases. Thickening of the pleural line with pleural line irregularity, focal, multifocal, confluent B lines, consolidations, air bronchograms, pleural effusion, and appearance of A lines during recovery phase may indicate COVID-19 pneumonia [29]. In our opinion, these procedures may be performed effectively and safely in experienced centers.

## 6. COVID-19 severity classification

There are several classifications in clinical use for the assessment of disease severity [30,31]. These classifications were developed for the evaluation of nonpregnant adult patients. However, they can be used in the pregnant population, too. The National Institutes of Health (NIH) classification is used in the United States [30]. It divides the disease into 5 groups: asymptomatic or presymptomatic infection, mild illness, moderate illness, severe illness, and critical illness according to the clinical findings [30]. Another classification from China categorizes patients into 3 groups: mild, severe, and critical [31]. According to the national guideline by the Turkish Ministry of Health, General Directorate of Public Health, patients are divided into 4 categories: uncomplicated cases, cases with mild/moderate pneumonia, cases with severe pneumonia, and critical cases that may necessitate intensive care unit (ICU) admission Turkish Ministry of Health, General Directorate of Public Health, COVID-19 Guideline, Scientific Committee Report: updated 21.08.2020. https://covid19bilgi.saglik.gov.tr/depo/rehberler/COVID-19_Rehberi.pdf?type=file [accessed on 31 May 2021]. The mentioned classifications are summarized in Table 1.

**Table 1:** Classifications for disease severity.

The National Institutes of Health (NIH) classification	
1) Asymptomatic or presymptomatic infection	Positive test for SARS-CoV-2 but no symptoms
2) Mild illness	Any signs and symptoms (e.g., fever, cough, sore throat, malaise, headache, muscle pain) without shortness of breath, dyspnea, or abnormal chest imaging
3) Moderate illness	Evidence of lower respiratory disease by clinical assessment or imaging and a saturation of oxygen (SaO2) ≥ 94% on room air at sea level
4) Severe illness	Respiratory frequency >30 breaths per minute, SaO2 < 94% on room air at sea level, ratio of arterial partial pressure of oxygen to fraction of inspired oxygen (PaO2/FiO2) < 300, or lung infiltrates > 50%
5) Critical illness	Respiratory failure, septic shock, and/or multiple organ dysfunction
Wu classification	
1) Mild illness	No or mild symptoms (fever, fatigue, cough, and/or less common features of COVID-19)
2) Severe illness	Tachypnea (respiratory rate >30 breaths per minute), hypoxia (oxygen saturation ≤ 93% on room air or PaO2/FiO2 < 300 mmHg), or >50% lung involvement on imaging)
3) Critical illness	Cases with severe clinical features like respiratory failure, shock, or multiorgan dysfunction
The National Guideline by Turkish Ministry of Health, General Directorate of Public Health	
1) Uncomplicated cases	a) Symptoms such as fever, muscle/joint pain, cough, sore throat, and respiration without distress (respiratory rate < 24, SpO2 > 93% on room air)ANDb) Patients with normal chest X-ray and/or lung tomography
2) Cases with mild/moderate pneumonia	a) Symptoms such as fever, muscle/joint pain, cough and sore throat, respiratory rate < 30/min, SpO2 > 90% on room airANDb) Mild-to-moderate pneumonia in chest radiography or tomography
3) Cases with severe pneumonia	a) Symptoms such as fever, muscle/joint pain, cough and sore throat, tachypnea (30/min), SpO2 below 90% on room airANDb) Bilateral diffuse pneumonia finding on chest radiography or tomography
4) Critical cases that may necessitate intensive care unit admission	Cases with at least one of the following clinical findings:a) Dyspnea and respiratory distressb) Respiratory rate ≥ 30/minc) PaO2/FiO2 < 300d) SpO2 < 90% or PaO2 < 70 mmHg despite 5 L/min oxygen therapye) Hypotension (systolic blood pressure < 90 mmHg and 40 mmHg from usual SBP and mean arterial pressure < 65 mmHg, tachycardia > 100/minf) Acute kidney injury, abnormal liver function test, development of acute organ dysfunction such as confusion, acute bleeding diathesis, and patients with immunosuppressiong) High troponin levels and arrhythmiah) Lactate > 2 mmoli) Skin disorders such as capillary return disorder and cutis marmaratus

COVID-19: Coronavirus disease 2019, SARS-CoV-2: Severe acute respiratory syndrome coronavirus 2

## 7. Course of COVID-19 in pregnancy

Current literature indicates that pregnancy does not increase the risk of acquiring COVID-19 but the course of disease seems to be worse in pregnant women compared to nonpregnant females of the same age [21,25,32–34]. Although more than 90% of pregnant women with COVID-19 recover without serious morbidity, rapid deterioration of disease may be observed. Especially symptomatic pregnant women have a higher risk for severe disease compared to the symptomatic nonpregnant women with COVID-19. Severe disease is more common in cases with coexisting comorbidities like obesity, asthma, older maternal age, hypertension, and diabetes [21,25,32–34]. Maternal death rates range between 0.14 and 0.80. However, due to the relatively low number of maternal mortality cases and the possible underestimation of asymptomatic cases, these rates may be deceptive [21,25,32–34]. According to the results of a report from the Centers for Disease Control and Prevention (CDC) including more than 23,000 pregnant women and over 386,000 nonpregnant females of reproductive age with symptomatic laboratory-confirmed SARS-CoV-2 infection, pregnant women with COVID-19 had higher risk for ICU admission, invasive ventilation, extracorporeal membrane oxygenation (ECMO), and mortality [25]. Another prospective study comparing 5183 pregnant women with 175,905 nonpregnant ones reported that pregnant women with COVID-19 had higher rates of mortality, pneumonia, and ICU admission [35]. 

## 8. Vertical transmission

The risk of vertical transmission has not been clearly revealed yet. According to a systematic review including neonates born to 936 infected pregnant women, neonatal PCR positivity was observed in 2.9% of the cases. In the same study, 1/34 cord blood samples and 2/26 placental samples were also positive for SARS-CoV-2. Finally, 3/82 neonatal serologies were immunoglobulin M (IgM)-positive for SARS-CoV-2 [36]. In utero transmission may occur by hematogenous or ascending route [37]. The theoretical risk for placental infection seems to be low since the coexpression of ACE-2 receptor and TMPRSS2 is minimal in the placental tissue [38]. However, SARS-CoV-2 can infect the placenta causing some histopathological changes [39]. This can be a contributing factor for COVID-19–related pregnancy complications. Another study from Turkey investigated the risk of maternal-fetal transmission in the early pregnancy and found no evidence of SARS CoV-2 infection in the fetal-placental samples [40]. The definitive diagnosis of congenital infection can only be made by the detection of SARS-CoV-2 RT-PCR positivity in umbilical cord blood or neonatal blood collected within the first 12 h of birth or amniotic fluid collected prior to rupture of membranes [41]. Transmission of SARS-CoV-2 can also occur in the intrapartum/postpartum period [42]. Thus, management of delivery and breastfeeding should be managed according to strict protocols in order to protect the neonates from the infection [23]. 

## 9. Obstetric and neonatal complications

According to the literature, COVID-19 may be associated with obstetric complications like preterm delivery, fetal distress and increased cesarean section rates [23,25,34,43–47]. It has been long known that excessive inflammation, hypoxia, maternal fever and worsening in maternal health condition may trigger premature labor and may cause fetal distress. However, obstetric complications may also occur in mild COVID-19 cases and more data is necessary to reach a clear conclusion. Moreover, most of the mentioned studies did not report iatrogenic prematurity rates. For this reason, information related to fetal distress and preterm delivery may be misleading [23,25,34,43–47]. Current data from the United States indicated an overall preterm delivery rate of 7.2% in pregnant women with COVID-19 compared to the ratio of 5.8% in pregnant women without COVID-19. However, the overall cesarean section rate was similar between the groups [48]. A current population-based study from England reported higher rates of fetal death and preterm delivery in patients with SARS-CoV-2 infection compared to pregnant women without SARS-CoV-2 infection. Furthermore, this study also reported increased risks for preeclampsia/eclampsia, emergency cesarean section, prolonged hospital admission after delivery, neonatal adverse outcome, and neonatal readmission in SARS-CoV-2 positive pregnant women [33]. 

The risk of miscarriage in pregnant women with COVID-19 is controversial. There is no direct evidence for increased miscarriage rates in infected pregnant women. On the other hand, there are concerns related to the possible risk of miscarriage especially in severe/critical cases [23,49–51]. Another important topic is the risk of congenital anomalies in pregnant women with COVID-19. However, no direct association has been reported between SARS-CoV-2 infection and increased rate of congenital anomalies for the time being [52–54]. Although overall stillbirth rate seems to be similar between SARS-CoV-2 positive and negative cases, an increased rate was reported for hospitalized patients [55,56].

Neonatal outcomes were generally favorable and more than 95% of the newborns were healthy according to the literature [25,57–60]. Majority of the neonates born to mothers with COVID-19 were asymptomatic. Symptomatic ones had symptoms of mild infection that did not require respiratory support [25,57–60]. The main reason behind the neonatal morbidity in infected pregnant women was prematurity most probably due to the increased rates of preterm deliveries performed severe COVID-19 [25,57–60].

According to the findings of a study from Turkey, 12.4% of the pregnant women with COVID-19 had pregnancy complications. Preterm delivery followed by miscarriage was the most common one. No cases of vertical transmission were observed in the mentioned study and neonatal intensive care unit (NICU) admission rate was 9.9% [23]. The roles of various cytokines, vitamins, mediators and trace elements on clinical outcomes were investigated in novel studies [61–64]. These studies indicated that the impact of disease on perinatal outcomes was associated with complex biological events and appropriate supplementation of some nutrients may improve the course of COVID-19 in selected cases [61–63]. 

The possible effect of COVID-19 on fetal Doppler parameters was also investigated in current studies [65,66]. No difference was present between healthy pregnant women and those with mild/moderate COVID-19 in terms of fetal Doppler parameters [66]. However, the pulsatility and resistance indices of umbilical and uterine arteries showed a significant increase in pregnant women recovered from COVID-19 compared to the controls [65]. The possible adverse effect of COVID-19 on the feto-placental circulation may be one of the factors behind the increased rates of perinatal complications in these cases. 

Another important topic is the effect of disease severity on obstetric complications [67–69]. These studies indicated that severe/critical disease was associated with increased rates of preterm and cesarean deliveries mostly due to the worsening in maternal health condition [67,68]. Moreover, a higher rate of obstetric complications was observed in cases with higher viral load [69].

## 10. Diagnosis

Nucleic acid amplification testing to detect SARS-CoV-2 RNA from the upper respiratory tract of the individuals is the most commonly used method for the definitive diagnosis of COVID-19 [70]. RT-PCR analysis of a nasopharyngeal swab specimen is the standard technique in Turkey [24]. A positive test confirms the diagnosis with a false negative rate ranging from 5% to 40%. A repeat test is recommended in cases with high clinical suspicion 24 to 48 h after the initial test. Lower respiratory specimens have higher sensitivity [71]. Although it is less sensitive than the nucleic acid amplification testing, antigen testing for SARS-CoV-2 may be preferred in selected cases with a rapid result [72]. Serologic testing may be used in the determination of previous SARS-CoV-2 infection or current infection with positive symptoms for 3 to 4 weeks [73]. Screening for clinical findings of COVID-19 should be performed in all patients admitted to a health-care facility. Performing a diagnostic test for all cases admitted to delivery may be considered according to the infrastructure of the hospitals. Special attention should be paid to differentiate COVID-19 from other respiratory pathogens and obstetric complications like preeclampsia with severe features Coronavirus infection and pregnancy Version 7 2020: updated 09/04/2020. Website https://www.rcog.org.uk/coronavirus-pregnancy [accessed on 31 May 2021].

## 11. Prenatal care

International organizations like The American College of Obstetricians and Gynecologists (ACOG), Royal College of Obstetrics and Gynecologists (RCOG), and the Society for Maternal-Fetal Medicine (SMFM) established their guidelines for the management of prenatal care during the COVID-19 pandemic [74]. Their recommendations mostly focus on decreasing the number of prenatal visits and shortening the time allocated for the examinations. Dividing the patients into two categories: low- versus high-risk patients (multiple gestation, hypertension, diabetes etc.) and decreasing the number of visits for low-risk population seem to be reasonable, active use of telemedicine services, limiting the number of persons in health-care settings, combining prenatal tests in the same visit, restricting visitors during the visits, providing a safe environment in healthcare facilities, strict hygiene control, and providing personal protective equipment during the visits are the main strategies to control the spread of disease according to current guidelines. Preferring 75 g 2-h oral glucose tolerance test and recommending cell-free DNA screening may help physicians to reduce the number of visits. Routine antenatal prophylactic corticosteroid therapy is recommended in cases with increased risk of preterm delivery. Low-dose aspirin (81 to 150 mg/day) can be administered safely in cases at high risk for preeclampsia. Patients with previous history of preeclampsia, multifetal gestation, chronic hypertension, diabetes mellitus, chronic kidney disease, obesity, or autoimmune disease with potential vascular complications have higher risk for developing preeclampsia. Low-dose aspirin for preeclampsia prevention should be initiated at ≥12 weeks of gestation, and ideally prior to 16 weeks in clinically indicated cases. The treatment can be continued until 36 weeks of gestation or 5 to 10 days before expected delivery time to decrease the risk of excessive postpartum bleeding [75]. Although there are some publications in the literature reporting increased risk of preeclampsia in pregnant women with COVID-19 routine administration of prophylactic aspirin is not recommended. Thus, clinicians should decide on the therapy based on clinical characteristics of the individuals [76]. The application of tocolysis is controversial and generally it is not recommended to delay delivery to administer antenatal steroids. Magnesium sulfate may be administered for the prophylaxis of eclampsia although it has a potential to cause respiratory muscle weakness. Prenatal invasive diagnostic tests may be performed if indicated [74]. Clinicians should also provide appropriate psychiatric support for the patients in this extraordinary period [77]. 

## 12. Hospitalization

Patients with progressive dyspnea, fever >39 °C despite antipyretics, intolerance for medications, persistent chest pain, confusion, obstetric complications, respiratory rate ≥20–24 /min, and/or heart rate >100 beats/min should be followed up in the hospital settings. Maternal peripheral oxygen saturation (SpO2) should be maintained at ≥95% [78]. Inpatient care indications are summarized in Table 2. 

**Table 2:** Inpatient care indications for pregnant women with COVID-19.

Presence of a comorbidity or obstetric complication (active uterine bleeding, preeclampsia, prelabor rupture of membranes, poor glycemic control in diabetic patients, uncontrolled hypertension etc.)
Fever > 39 °C despite use of acetaminophen
Moderate/severe symptoms (oxygen saturation < 95% on room air, respiratory rate > 30/min, rapidly increasing need for supplemental oxygen)
Critical COVID-19 (Respiratory failure, hypotension despite appropriate hydration, and/or new end-organ dysfunction)

COVID-19: Coronavirus disease 2019

## 13. Medical therapy for COVID-19 in pregnant women

New medication alternatives are being proposed every day for the treatment of COVID-19, and our knowledge about the use of most of these drugs in pregnancy is limited. Below we discussed some of the most popular medications in brief.

### 13.1. Anticoagulants

The risk of venous thromboembolism was found to be increased in pregnant women with COVID-19 [48]. Infection with SARS-CoV-2 should be considered a transient risk factor for venous thromboembolism. Prophylactic-dose anticoagulation is recommended for hospitalized patients with severe COVID-19. If thromboprophylaxis has been started in a self-isolated patient, it should be continued until recovery from acute illness (between 7 and 14 days). The optimal dose, duration, and type of thromboprophylaxis should be chosen by a multidisciplinary team in severe/critic cases. Thromboprophylaxis should be continued for 10 days following hospital discharge and longer duration of thromboprophylaxis may be considered for patients with serious morbidity. Thromboprophylaxis should be offered for all pregnant women admitted with confirmed or suspected COVID-19, unless birth is expected within 12 h or there is significant risk of hemorrhage. For women within 6 weeks of their postpartum period, thromboprophylaxis should be administered for the duration of their admission and for at least 10 days after discharge. Thromboprophylaxis may be extended until 6 weeks postpartum in cases with serious morbidity. Low-molecular-weight heparin (enoxaparin 40 mg subcutaneously every 24 h) or unfractionated heparin (5000 units in the first trimester, 7500 to 10,000 units in the second trimester, and 10,000 units in the third trimester, administered subcutaneously every 12 h) may be used for thromboprophylaxis. Laboratory parameters like D-dimer, ferritin, and CRP may be helpful for the clinicians for ongoing of anticoagulants after recovery of COVID-19 symptoms on pregnant women Coronavirus infection and pregnancy Version 7 2020: updated 09/04/2020. Website https://www.rcog.org.uk/coronavirus-pregnancy [accessed on 31 May 2021]. Intermittent pneumatic compression may be performed when anticoagulant therapy is contraindicated [79]. 

### 13.2. Glucocorticoids

Dexamethasone 6 mg/day for 10 days or until discharge may be considered for severe cases that necessitate supplemental oxygen or ventilatory support. Glucocorticoid therapy may also be administered in critical cases with refractory shock. As dexamethasone crosses the placenta, it also has a favorable effect on fetal lung maturation in preterm labor. Other steroids like methylprednisolone or hydrocortisone may be used when less fetal exposure is preferred [80]. 

### 13.3. Nonsteroidal antiinflammatory drugs

Nonsteroidal antiinflammatory drugs may cause unfavorable adverse effects on the fetus like oligohydramnios, premature closure of the ductus arteriosus. For this reason, they should be administered in the lowest dose possible and the treatment should not be continued more than 48 hours [81]. Acetaminophen is considered a safe alternative in pregnant patients; however, there are concerns about its use in cases with elevated liver enzymes [82].

### 13.4. Hydroxychloroquine

The immunomodulatory and anti-inflammatory effects of hydroxychloroquine have long been known. Although its effect in SARS-CoV-2 infection has not been clearly identified yet, modification on ACE-2 glycosylation and increase in the pH of the endosomes are thought to be the possible mechanisms. However, physicians should be cautious about the potential risk of ventricular arrhythmias [83]. Although it crosses the placenta and is excreted in breast milk, no adverse effect on the fetus has been observed in the observational studies [84]. 

### 13.5. Azithromycin

It is a macrolid antibiotic with potential antiviral, immunomodulatory, and antiinflammatory characteristics. Although its role in the treatment of COVID-19 is questionable, it elevates cellular pH and disrupts the binding of SARS-CoV-2 with ACE-2 receptors. Moreover, it has some immunomodulatory properties which slows down cytokine storm. However, physicians should be cautious about the prolongation of the QT interval. It is regarded as category B for administration during pregnancy. Initial dose of 500 mg per oral followed by 250 mg per oral daily for 4 days may be administered for the treatment of COVID-19 [85]. 

### 13.6. Lopinavir–ritonavir

This combination drug consists of two antiretroviral protease inhibitors primarily used for human immunodeficiency virus (HIV). However, it may be used for the treatment of SARS-CoV-2 infection although there are ongoing debates [86]. It is considered category C for pregnant women and no potential adverse effect was observed in pregnant women with HIV [87]. 

### 13.7. Remdesivir

It is a novel nucleotide analog used for the treatment of SARS-CoV-2. No fetal toxicity was reported for its use in Ebola and Marburg viruses [88]. 

### 13.8. Favipiravir

It is an antiviral, nucleoside analog that inhibits RNA-dependent RNA polymerase. It was mainly used for oseltamivir-resistant influenza viruses but it is regarded as a potential medication for SARS-CoV-2 infection with favorable outcomes. However, there is insufficient data regarding its use in pregnant and lactating women. Thus, its application should be avoided in these specific populations [89].

### 13.9. Tocilizumab

It is a monoclonal antibody that inhibits IL-6. It has been mainly used for rheumatologic diseases. As IL-6 is one of the main actors of cytokine release syndrome, administration of tocilizumab may be beneficial in severe patients with COVID-19. However, its safety in pregnant and lactating women is questionable. As it can be excreted in breast milk, lactation should be avoided in patients taking this medication [84,87].

### 13.10. Anakinra

It is an IL-1 receptor antagonist that has a potential to block the proinflammatory cytokine cascade. Although it has been primarily used for rheumatologic diseases, some studies indicated favorable results in COVID-19 cases. It may be used in pregnant women although our knowledge is still limited [84,87].

### 13.11. Convalescent plasma

Although convalescent plasma has been used in some pregnant women with COVID-19, its efficacy is controversial. Thus, it is not a routine part of therapy for pregnant population [90]. 

### 13.12. Neutralizing monoclonal antibodies

Combination of monoclonal antibodies like Bamlanivimab-etesevimab or casirivimab-imdevimab may be used for the treatment of SARS-CoV-2 infection. They can be used in cases with mild/moderate disease with a potential for rapidly progressing to severe/critical disease. They have a potential to cross the placenta and affect the fetus. However, our knowledge is insufficient regarding their impact on the developing fetus [89].

## 14. Vaccines

Pregnancy is considered a risk factor for severe COVID-19 by healthcare authorities [6,7]. Thus, protecting pregnant women from SARS-CoV-2 infection is crucial to decrease maternal morbidity and mortality. Vaccination seems to be the most promising method to control the spread of COVID-19, and there are various vaccine platforms that have been administered on large populations in the last months. However, due to the exclusion of pregnant/lactating women from the preliminary vaccine trials, our knowledge is limited on the safety and efficacy of vaccines in these vulnerable populations [91]. Most preapproved vaccines work by introducing an antigen into the body to induce an immune response. The antigen can be an inactivated infectious agent (Sinovac, Sinovac Biotech, China) or a protein purified from the infectious agent. In contrast, COVID-19 mRNA vaccines developed by Pfizer-BioNTech (BNT162b2, Pfizer-BioNTech, Germany) and Moderna (mRNA-1273, ModernaTX, the United States) work by carrying the genetic information required to produce the spike protein of SARS-CoV-2, the protein found on the virus surface. When the vaccine is injected into muscle cells, they produce the spike protein recognized by the immune system, the mRNA never enters the nucleus and therefore does not integrate into the DNA; within hours to days, mRNA is degraded in the cell cytoplasm. Vaccines developed by AstraZeneca–Oxford (AZD1222, AstraZeneca-Oxford, the United Kingdom) and Janssen-Johnson and Johnson (Ad26.COV2.S, Janssen-Johnson and Johnson, the United States) use a modified viral vector to deliver the spike protein of SARS-CoV-2 to cells, which then trigger an immune response. The AstraZeneca-Oxford (AZD1222, AstraZeneca-Oxford, the United Kingdom) vaccine uses a nonreplicable modified chimpanzee adenovirus, whereas the Janssen-Johnson and Johnson vaccine (Ad26.COV2.S, Janssen-Johnson and Johnson, the United States) uses Human Adenovirus 26, a nonreplicating modified human adenovirus. Vaccines developed by Novavax (Novavax, the United States) and GSK-Sanofi (VAT00008, GSK-Sanofi, France-the United Kingdom) are protein subunit vaccines in which a baculovirus is used to produce the recombinant protein in insect cells. Both of these vaccines have been mixed with adjuvants to boost the immune response [92]. As none of the vaccine platforms contains live viral particles which have a potential to replicate, they are considered theoretically safe in pregnancy. However, adverse events may occur due to altered immune response [93]. Preliminary data from a limited number of studies demonstrated no adverse events in vaccinated pregnant women and they reported transfer of maternal antibodies across the placenta and into breast milk [94,95]. Among 3958 participants enrolled in the v-safe pregnancy registry, 827 had a completed pregnancy and no prominent adverse event was reported [94]. Thus, according to the preliminary data, vaccination may be offered for pregnant women as they have an increased risk for severe disease. ACOG recommends vaccination for all eligible pregnant and lactating women ACOG. Novel Coronavirus 2019: updated 1.5.2021. Website https://www.acog.org/clinical/clinical-guidance/practice-advisory/articles/2020/03/novel-coronavirus-2019 [ accessed on 31 May2021]. Both Turkish Society of Obstetrics and Gynecology and Maternal-Fetal Medicine and Perinatology Society of Turkey have recommended vaccination during pregnancy. They stated that pregnant women could be vaccinated safely after the first trimester (14 weeks of pregnancy) with their rappel intervals. Either inactivated or mRNA vaccine platforms may be preferred according to mentioned societies Turkish Society of Obstetrics and Gynecology: Committee Opinion on The Administration of COVID-19 Vaccines During Pregnancy and in the Postpartum Period. Website https://www.tjod.org/gebelik-ve-dogum-sonrasi-donemde-covid-19-asilari-ile-ilgili-tjod-gorusu/ [accessed on 31 May 2021], Maternal-Fetal Medicine and Perinatology Society of Turkey: Committee Opinion on The Administration of COVID-19 Vaccines During Pregnancy and in the Postpartum Period. Website https://www.tmftp.org/files/uzman-gorusleri/gebelerde_covid19_asisi.pdf [accessed on 31 May 2021]. 

## 15. Labor, delivery, and postpartum care of pregnant women with COVID-19

Timing of delivery should be decided based on maternal health condition, accompanying obstetric complications and gestational age [4,23]. For asymptomatic cases and patients with nonsevere COVID-19, delivery at <39 weeks of gestation should be avoided unless there is an obstetric complication that necessitates prompt delivery [4,23]. For severe/critical cases, an individualized approach should be preferred. Delivery may be considered at >32–34 weeks of gestation for severe not intubated cases. The management of intubated cases should be performed according to maternal clinical characteristics. Prompt delivery may be considered in the presence of refractory hypoxemic respiratory failure or worsening critical illness [4,23].

Strict precautions should be taken to reduce the risk of viral transmission during delivery. Screening all patients for clinical manifestations of COVID-19, using adequate personal protective equipment, providing single-occupancy rooms with good ventilation and reducing the number of persons in the delivery room are the primary steps for the healthcare facilities. Diagnostic tests may be performed for scheduled labor inductions and cesarean deliveries. Contact and droplet precautions should be followed for the delivery of confirmed or highly suspected cases [4,23]. A novel delivery table shield has been used during the vaginal delivery of suspected/confirmed cases in our institution since the early days of the pandemic. This equipment protects the healthcare professionals from respiratory droplets during the second stage of labor allowing eye contact and comfortable respiration for the mother [96]. The demonstration of the mentioned delivery table shield is shown in Figure.

**Figure:**
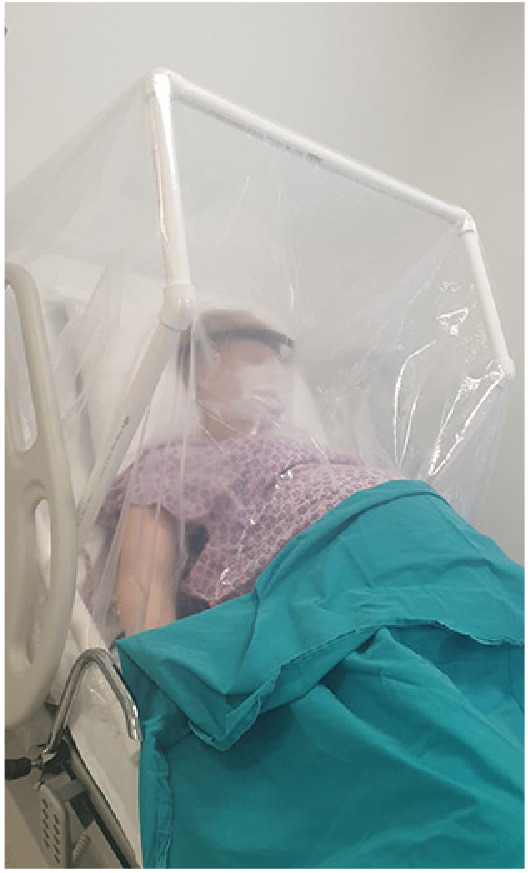
The delivery table shield used in our institution during the vaginal delivery of suspected/confirmed cases with COVID-19.

Cesarean delivery should be performed for obstetric indications. However, acute decompensation in critical cases may also necessitate prompt delivery with cesarean section [4,23]. Induction of labor may be performed for intubated patients but follow-up of labor may be difficult in the ICU settings. Cesarean section may be associated with an increased risk for clinical worsening but more data is necessary to achieve more precise results on this issue [97]. 

Regional anesthesia is generally recommended in the delivery of pregnant women with COVID-19 due to its advantages for both the patient and the healthcare staff. Use of nitrous oxide is controversial and most of the authorities recommend not to use it [98]. 

Continuous electronic fetal monitoring is recommended during labor. Although SARS-CoV-2 is rarely detected in amniotic fluid, vaginal secretions, and feces, there is a potential risk for transmission especially during the second stage of labor. Thus, necessary measures should be taken to decrease the risk of transmission during delivery. Disinfection with ultraviolet light ≥60 min with at least 30 min of ventilation following irradiation should be provided after each delivery to decrease the risk of viral transmission [4,23]. 

Skin-to-skin contact between the mother and the neonate should be supported in the delivery room providing necessary precautions like strict hand hygiene and wearing a mask [99]. Procedures like delayed cord clamping or cord blood banking may be performed if necessary. Management of postpartum hemorrhage should be performed according to current guidelines. However, physicians should be cautious while using tranexamic acid and methylergometrine as these drugs may aggravate COVID-19–related complications like thrombosis and vasoconstriction. Prophylactic-dose anticoagulation is recommended for postpartum patients with severe/critical COVID-19. Rooming-in should be provided and breast feeding should be encouraged as long as necessary precautions for viral transmission are taken [4,23,99]. 

## 16. Conclusion

In conclusion, the course of COVID-19 during pregnancy is generally mild. However, SARS-CoV-2 infection in pregnant women may rapidly progress into severe disease and increased rates of obstetric complications are observed in these cases. An individualized approach should be provided by a multidisciplinary team for the management of pregnant women with COVID-19 to achieve favorable outcomes. Preliminary results are promising for the administration of SARS-CoV-2 vaccines in the pregnant population. Special protocols should be followed to prevent the transmission of SARS-CoV-2 during the labor and delivery. 
